# Causal insights into the school-family-research integrated health promotion program for overweight and obesity: the independent role of moderate-to-vigorous physical activity in body fat reduction, undermined by psychological factors

**DOI:** 10.3389/fnut.2025.1577319

**Published:** 2025-07-29

**Authors:** Xuehui Zhang, Xiang Pan, Bing Liu, Yibo Gao, Lupei Jiang, Xiaoxiao Chen, Deqiang Zhao, Yibei Wang, Haixia Hu, Xueli Zhao, Jiahui Lu, Koya Suzuki, Yanfeng Zhang

**Affiliations:** ^1^College of Sports Science, Hefei Normal University, Hefei, China; ^2^China Institute of Sport Science, Beijing, China; ^3^Graduate School of Health and Sports Science, Juntendo University, Inzai, Japan; ^4^School of Literature and Journalism, Yantai University, Yantai, China; ^5^College of Physical Education and Sports Rehabilitation, Jinzhou Medical University, Jinzhou, China; ^6^Weifang Aiyoudong Children and Youth Sports Health Research Institute, Weifang, China; ^7^Department of Psychology, Qingdao University, Qingdao, China; ^8^Institute of Health and Sports Science & Medicine, Juntendo University, Inzai, Japan

**Keywords:** adolescent, obesity, physical fitness, cross-lagged model, body composition, exercise self-efficacy, self-esteem, physical activity

## Abstract

**Introduction:**

This study implements the School-Family-Research Integrated Health Promotion Program for Overweight and Obesity (SFR-OO), which combines exercise and dietary interventions to combat adolescent obesity. It aims to enhance body composition, exercise motivation, SE, and physical fitness. By using a cross-lagged model, the study will explore causal relationships between self-esteem (SE), exercise self-efficacy (ESE), physical activity, and body composition.

**Methods:**

Ninety-eight adolescents were randomly assigned to either the intervention group or the control group. The intervention group received the SFR-OO intervention for 12 weeks. Assessments included physical fitness tests, body composition, and psychological indicators.

**Results:**

While both groups showed a time effect (*p* < 0.001), the intervention resulted in a significant decrease in body fat percentage (BFP) compared to the control group (*p* < 0.001, *p* < 0.038). Improvements were greater for knee push-ups, standing long jump, 4 x 10 meters round trip run and supine trunk raise time as well as psychometric measures (*p* < 0.001). In the intervention group, higher baseline MVPA significantly predicted greater reductions in BFP at 12 weeks (*β* = −0.169), whereas no such predictive relationship was found in the control group.

**Discussion:**

The 12-week SFR-OO effectively enhanced adolescents’ body composition, physical fitness, and psychological outcomes. However, SE and ESE did not significantly predict MVPA or BFP. MVPA modestly predicted reduced BFP only within the intervention group, suggesting a context-dependent effect. This study was registered with ClinicalTrials.gov under the registration number NCT06524908.

## 1 Introduction

The rise in obesity is now a global health issue. It affects the health, academic performance, and mental growth of teenagers. Obese teens often feel less satisfied with their body image (1). This affects their self-esteem (SE) (2) and harms their mental health and social abilities.

Increasing physical activity is crucial for dealing with obesity. Some studies (3) have shown a link between Exercise self-efficacy (ESE) and physical activity. ESE (4) means believing in one’s ability to complete exercise tasks. It affects how teens start and stick to physical activity. High SE (5) and ESE (6) help teens manage body composition. In overweight adults, exercise programs boost ESE (7). But, not much research focuses on how ESE and physical activity connect in teens. Most studies feature single variables or cross-sectional designs. They lack clear proof of causal links and miss the complex interplay of psychological factors, physical activity, and body fat.

Family and school are key settings for teens. Teamwork between schools and families is vital for teen development (8). Yet, barriers exist like poor communication and management issues (9). There is also little evidence on health promotion among guardians (10) and school staff (11). This highlights the need for collaboration between researchers and experts.

This study put in place a School-Family-Research Integrated Health Promotion Program for Overweight and Obesity (SFR-OO), aiming to improve collaboration between schools and families. Researchers provided theoretical guidance and monitored implementation. The goals included optimizing body composition, boosting exercise motivation and self-esteem (SE), and improving physical fitness. Unlike traditional interventions that often focused on only one setting (such as school-based or family-based programs), SFR-OO integrated school administrators, student participants, their guardians, and ongoing supervision and education from research staff. This approach served as a regional demonstration project, piloting the transformation of scientific research into community health welfare through joint management, continuous monitoring, and timely feedback among multiple stakeholders. We assessed SFR-OO through these metrics and applied a cross-lagged model. This enabled us to explore the causal links among SE, exercise self-efficacy (ESE), physical activity, and body composition, thereby providing scientific evidence for improved adolescent obesity management.

The aim of this study was to evaluate the effectiveness of SFR-OO in improving body composition, exercise self-efficacy, self-esteem, and physical fitness among overweight and obese adolescents, and to investigate the potential causal relationships among these factors. We hypothesized that the intervention would improve body composition, increase ESE and SE scores, enhance physical fitness, and that there would be causal links among ESE, SE, moderate-to-vigorous physical activity (MVPA), and changes in body fat percentage (BFP).

## 2 Methods

### 2.1 Sample size calculation

Recent meta-analyses have demonstrated that the effect sizes (Cohen’s *d*) for physical activity interventions on key outcomes such as body fat percentage, self-esteem, and exercise self-efficacy in adolescents are fairly comparable, generally ranging from 0.22 to 0.32 (12–14). Based on the lowest observed effect size (*d* = 0.22), we used G*Power 3.1 to estimate the required sample size, with parameters set at *α* = 0.05, power (1-*β*) = 0.80, and a pre-post correlation of 0.80. Under both paired and independent samples t-test frameworks, the minimum sample size was approximately 40 participants per group. Allowing for potential attrition, we pragmatically set a target of 50 per group (total n = 100) to ensure adequate power for detecting effects in our main outcomes.

For our main analyses employing a two-variable, two-wave cross-lagged panel model (CLPM), recent methodological work—including Monte Carlo simulations—suggests that a total sample size of 80 to 100 is usually sufficient to achieve stable parameter estimation and adequate statistical power, especially when the number of estimated parameters is limited (15). Furthermore, Sim et al. (16) have shown through extensive Monte Carlo simulation that, for path models with all observed variables (which are structurally analogous to two-variable CLPMs), a sample size of 50 is adequate to meet criteria for parameter bias, 95% confidence interval coverage, and statistical power, provided the effect size is medium (ab ≥ 0.13–0.26) or larger.

### 2.2 Procedure

**Figure 1:**
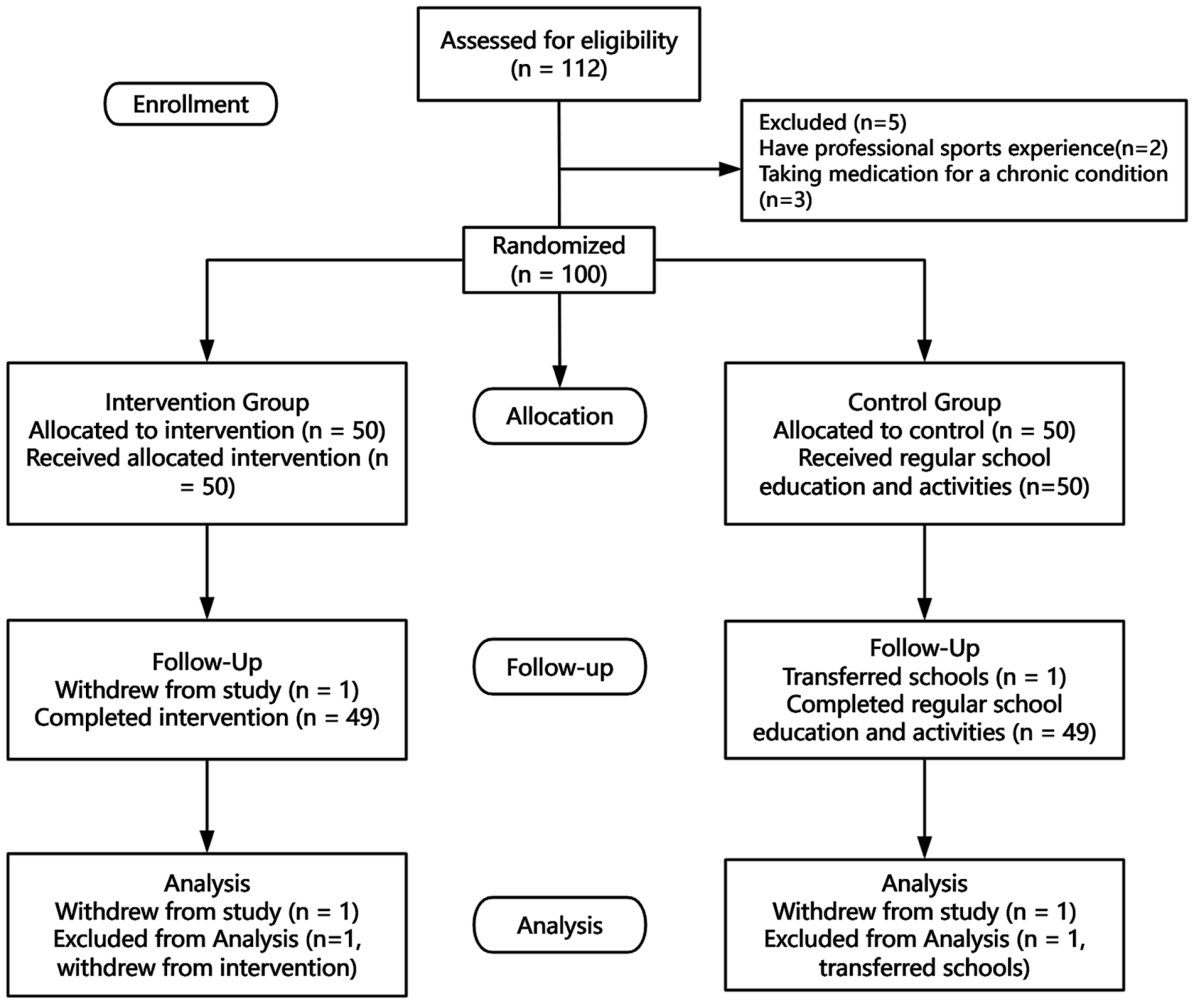
Consort flow diagram.

We recruited participants from August 10 to September 1, 2022. Initial health screenings were conducted, with inclusion criteria as follows: (1) age between 13 and 15 years (chosen to facilitate standardized management and intervention delivery within the school); (2) assessed as physically healthy through the Physical Activity Readiness Questionnaire (PAR-Q); (3) no history of professional athletic experience; (4) ability to comprehend the testing procedures, voluntary participation in the entire assessment process, and provision of informed consent. Exclusion criteria included: (1) severe organic lesions of the cardiovascular, neurological, pulmonary, renal, or musculoskeletal systems; (2) ongoing medication for chronic illnesses; (3) history of mental illness; (4) inability to complete follow-up or demonstrated poor compliance. We randomly assigned 100 adolescents to either the intervention or control group. All participants completed baseline outcome measurements before the intervention. The intervention group received a 12-week intervention. The control group continued with regular school education and activities without any specific intervention. They maintained their usual lifestyle. After the 12 weeks of intervention, all interventions were stopped. Following this, all participants underwent post-intervention testing. (Figure 1).

### 2.3 School-family-research integrated health promotion program for overweight and obesity

We designed the resistance training to allow a 72-h rest period for each muscle group (17). The program followed a three-day split routine: anterior upper body, posterior upper body, and lower body. Each major muscle group had targeted resistance exercises. Participants could adjust intensity based on their ability, using regressions or progressions. The exercises included: (1) Squats for the lower body; (2) Crunches for the trunk; (3) Push-ups for the upper body; (4) Nordic drops for the lower body flexors; (5) Prone back extensions for the trunk; (6) Pull-ups for the upper body; (7) Shoulder press for the upper body; (8) 50 m sprints for overall lower body. The intensity was set at 70–80% of 1RM (one-repetition maximum), with a target of 8–12 reps using bodyweight resistance. Regression was used if participants did fewer than 7 reps, while progression was applied if they did 13 or more. Each exercise had four sets, with 8–12 reps per set and 60 s rest between sets. Each session lasted 30 min and occurred between 4:30 PM and 5:00 PM. Every 4 weeks, the 1RM was tested to adjust the load and ensure progress. For resistance bands, intensity was calculated with: 1RM = Resistance × Reps/30 + Resistance (18). A comprehensive overview of each weekly training program, specific exercises, session durations, equipment used, repetitions, and progression criteria is provided in Supplementary materials 1.

The aerobic program used maximum heart rate (MHR) to set intensity. The formula was: MHR = 206.9 - (0.67 × Age). Training occurred at 57–67% of each participant’s MHR (19). This range supports cardio health and fat oxidation. Participants monitored their heart rate with fitness trackers, receiving real-time feedback to ensure they remained within the target range. This strategy enhanced training precision and allowed for immediate adjustments. Training sessions were 30 min long and happened between 5:00 PM and 5:30 PM.

Given certain participant constraints, we used a semi-flexible dietary intervention. The Harris-Benedict equation helped calculate total daily energy expenditure (TDEE) (20). We restricted daily intake to 75% of TDEE. Parents recorded their child’s dietary intake, including total calories and types of food consumed. We regularly reviewed these records to monitor compliance. Parents received training on “Boohe Health” calorie tracking software. Dietary assessments took place every 2 weeks over 4 days, covering three weekdays and one weekend. Parents filled out 24-h dietary recall forms.

### 2.4 Indicators assessment

Physical Fitness: The physical fitness assessments in this study included the 50-meter sprint, standing long jump, 4 × 10-meter shuttle run, knee push-ups, plank, supine trunk lift time, vital capacity, and relative vital capacity. The 50-meter sprint and standing long jump were conducted according to the National Standards for Students’ Physical Health (2014 revision) (21). The 4 × 10-meter shuttle run, knee push-ups, and supine trunk lift time were measured following the protocols of the Eurofit Physical Fitness Test Battery (22). Notably, the knee push-up was implemented as a youth-adapted modification of the original Eurofit push-up test, while the supine trunk lift time was also based on the Eurofit supine trunk lift, but the measured outcome was changed from maximal elevation height (primarily reflecting flexibility and static strength) to the maximum duration the subject could maintain the raised position (reflecting trunk extensor endurance). Detailed procedures and scoring criteria for these modified tests are provided in Supplementary materials 1. The plank test was performed in accordance with the standardized core endurance protocol described by McGill et al. (23). Vital capacity and relative vital capacity were assessed using an electronic spirometer.

Body Composition: Body composition measurements included height, body mass, BMI, BFP, fat mass (FM), and fat-free mass (FFM). Height and body mass were measured using an electronic stadiometer and weighing scale, and BMI was calculated as body mass (kg) divided by height squared (m^2^). BFP, FM, and FFM were assessed using a multi-frequency bioelectrical impedaS1nce analyzer (InBody 3.0, South Korea), with all measurements performed according to standardized procedures outlined in the device manual.

Psychological Indicators: The definition of SE utilized in this study is primarily based on the widely accepted framework proposed by Rosenberg, which posits SE as an individual’s positive or negative attitude toward self. The SE Scale is an evaluation tool designed to measure an individual’s SE psychological state, originally developed by American psychologist Morris Rosenberg in 1965 (24). The measurement of ESE was conducted using a validated ESE questionnaire (25). This questionnaire is a reliable and effective instrument for assessing individuals’ confidence in their exercise capabilities and can be utilized to evaluate self-efficacy in exercise behavior (4, 26).

Physical Activity Level: MVPA refers to physical activities conducted at a moderate intensity or higher. The Actigraph accelerometer provides a more precise assessment of an individual’s activity level by detecting variations in acceleration (27). Participants were instructed to wear the Actigraph GT3X + securely on their left wrist to ensure proper fit. They were required to wear the accelerometer throughout the day and record a minimum of 4 days of activity, including at least one weekend day, for the data to be included in the analysis. The calculation for MVPA time was as follows: [(average weekday MVPA * 5) + (average weekend MVPA * 2)]/7.

### 2.5 Statistical analysis

In this study, we performed statistical analyses using R version 4.3.2. Normality tests were conducted to determine the appropriate method for subsequent difference testing. For non-normally distributed data, we reported the median (P25, P75). The Mann–Whitney U test was used to compare between-group differences in intervention effects, while the Wilcoxon signed-rank test was used to evaluate within-group changes from pre- to post-intervention. To assess the longitudinal effects of time and the intervention on outcome variables, and to control for potential confounding effects of sex and age, generalized linear regression models were constructed separately for each outcome variable, with time and the intervention as independent variables, a log link function, and a Poisson distribution for the dependent variable. We constructed cross-lagged models in AMOS to analyze longitudinal predictive relationships, running separate models for the intervention and control groups.

### 2.6 Declaration

This study was registered with ClinicalTrials.gov under the registration number NCT06524908 (01/08/2022). Ethical approval was obtained from the Ethics Committee of China Institute of Sport Science, No. CISSLA20220801. All study participants signed informed consent forms, ensuring the confidentiality of research data. All research involving humans was carried out in accordance with The Code of Ethics of the World Medical Association (Declaration of Helsinki).

## 3 Results

### 3.1 Descriptive statistics

Initially, 100 participants were enrolled: 50 in the Intervention Group (25 M, 25F) and 50 in the Control Group (24 M, 26F). During the study, one F from the Intervention Group and one M from the Control Group withdrew. Thus, 98 completed the study: the Intervention Group had 49 (25 M, 24F) and the Control Group had 49 (23 M, 26F).

### 3.2 Comparison of before and after intervention

Both the intervention and control groups showed significant time effects on body weight, FFM, and BMI (*p* < 0.001) (Table 1). This means these indicators changed over time. However, no significant group or group-by-time interaction effects were found. Both groups showed significant changes over time in FM (*p* < 0.001). But again, there were no significant group differences or interactions. The FFMI also changed over time in both groups (*p* < 0.001), but the intervention had no significant effect. BFP significantly decreased over time in the intervention group (*p* < 0.001). In the control group, it significantly increased (p < 0.001). After the intervention, BFP was significantly lower in the intervention group than in the control group (*p* = 0.038); no significant difference was found at baseline (*p* = 0.299). There was a significant interaction between the group and time (*p* = 0.017). Fifty minutes sprint times changed over time in both groups (*p* < 0.001), but there were no significant group differences or interactions. The plank time showed a significant time effect in the control group (*p* < 0.001), but not in the intervention group (*p* = 0.356). There were no significant group differences or interactions. Knee push-ups, standing long jump, 4x10m shuttle run, and supine trunk lift time all showed significant time effects for both groups (*p* < 0.001). However, the intervention group improved significantly more than the control group (*p* < 0.001). There was a significant interaction between the intervention and time (*p* < 0.001). Both groups showed significant time effects for vital capacity and relative vital capacity (*p* < 0.001). Yet, the intervention had no significant effect on vital capacity. The relative vital capacity showed no significant group difference, but there was a significant interaction with time (*p* = 0.011). ESE and SE scores increased significantly in both groups over time (*p* < 0.001), with greater improvements observed in the intervention group (*p* < 0.001) and a significant intervention-by-time interaction (*p* < 0.001).

**Table 1:** Changes in outcome indicators before and after the intervention.

Variable		**Treatment**	**Median (P25, P75)**			**Generalized linear regression**		
			**T1**	**T2**	***P* (T1 vs. T2)**	**Term**	**Estimate**	***P***
Body composition	Body weight (kg)	Intervention	59.8 (50.7, 74.2)	58.6 (49.3, 73.9)	<0.001	Treatment	−5.16	0.4
		Control	60.6 (51.0, 69.3)	61.3 (51.7, 70.1)	<0.001	Time	−5.47	0.373
		P (I vs. C)	0.488	0.728		Treatment × time	3.32	0.393
	Fat free mass (kg)	Intervention	44.0 (38.4, 51.5)	44.1 (38.5, 52.0)	<0.001	Treatment	−0.667	0.868
		Control	44.2 (40.0, 48.0)	44.4 (40.0, 48.1)	0.572	Time	0.145	0.971
		P (I vs. C)	0.774	0.755		Treatment × time	0.0898	0.972
	Fat mass (kg)	Intervention	16.8 (13.7, 25.0)	14.9 (12.1, 21.2)	<0.001	Treatment	−4.5	0.091
		Control	16.3 (11.5, 20.7)	17.2 (12.8, 20.9)	<0.001	Time	−5.62	0.035
		P (I vs. C)	0.238	0.295		Treatment × time	3.23	0.056
	Body fat percentage (%)	Intervention	28.2 (25.0, 32.7)	26.2 (22.0, 28.4)	<0.001	Treatment	−4.63	0.056
		Control	26.8 (23.5, 31.7)	28.2 (24.5, 32.2)	<0.001	Time	−6.41	0.008
		P (I vs. C)	0.299	0.038		Treatment × time	3.65	0.017
	BMI (kg/m ^2^)	Intervention	22.72 (20.14, 26.52)	21.71 (19.32, 25.06)	<0.001	Treatment	−1.73	0.338
		Control	22.82 (19.15, 25.68)	23.25 (19.82, 26.04)	<0.001	Time	−2.09	0.248
		P (I vs. C)	0.502	0.506		Treatment × time	1.21	0.291
Psychological indicators	Exercise self-efficacy (score)	Intervention	33 (30, 40)	43 (40, 53)	<0.001	Treatment	12.9	0.002
		Control	33 (32, 40)	37 (33, 43)	0.058	Time	23	<0.001
		P (I vs. C)	0.404	<0.001		Treatment × time	−10.8	<0.001
	Self-esteem (score)	Intervention	22.8 (19.4, 25.9)	32.1 (27.4, 37.0)	<0.001	Treatment	9.96	<0.001
		Control	24.9 (21.4, 27.5)	25.1 (21.3, 30.2)	0.171	Time	16.8	<0.001
		P (I vs. C)	0.051	<0.001		Treatment × time	−8.21	<0.001
Physical activity level	MVPA (min/week)	Intervention	52.76 (48.68, 55.96)	60.32 (54.00, 64.88)	<0.001	Treatment	7.88	0.008
		Control	52.32 (48.08, 54.88)	51.36 (47.20, 54.16)	0.001	Time	15.3	<0.001
		P (I vs. C)	0.912	<0.001		Treatment × time	−8.11	<0.001
Physical fitness	50 m Sprint (s)	Intervention	11.43 (9.73, 12.87)	11.36 (9.41, 13.12)	0.145	Treatment	−0.705	0.349
		Control	11.86 (10.59, 13.12)	12.44 (11.02, 14.30)	<0.001	Time	−1.01	0.183
		P (I vs. C)	0.449	0.026		Treatment × time	0.857	0.073
	Plank (s)	Intervention	45.41 (34.81, 59.00)	45.76 (35.53, 56.74)	0.588	Treatment	5.96	0.584
		Control	40.16 (32.26, 67.45)	34.14 (28.49, 58.71)	<0.001	Time	7.59	0.485
		P (I vs. C)	0.527	0.101		Treatment × time	−7.09	0.303
	Knee push-ups (reps)	Intervention	9 (6, 16)	16 (9, 28)	<0.001	Treatment	10.8	0.006
		Control	10 (6, 17)	9 (5, 16)	<0.001	Time	19	<0.001
		P (I vs. C)	0.533	<0.001		Treatment × time	−10.1	<0.001
	Standing long jump (m)	Intervention	1.34 (1.18, 1.55)	1.41 (1.22, 1.58)	<0.001	Treatment	0.114	0.259
		Control	1.27 (1.14, 1.45)	1.15 (1.03, 1.31)	<0.001	Time	0.24	0.018
		P (I vs. C)	0.177	<0.001		Treatment × time	−0.182	0.005
	4 × 10-m shuttle run (s)	Intervention	18.06 (15.04, 22.78)	17.56 (14.08, 20.90)	0.017	Treatment	−2.03	0.091
		Control	18.00 (14.88, 20.76)	20.46 (17.02, 24.08)	<0.001	Time	−2.34	0.051
		P (I vs. C)	0.977	0.009		Treatment × time	1.66	0.029
	Supine trunk lift time (s)	Intervention	15.31 (10.87, 20.33)	31.91 (26.41, 38.05)	0.033	Treatment	15.3	0.002
		Control	15.58 (11.89, 24.16)	16.39 (11.90, 25.96)	<0.001	Time	31.9	<0.001
		P (I vs. C)	0.685	<0.001		Treatment × time	−15.5	<0.001
	Vital capacity (ml)	Intervention	2,774 (2,317, 3,154)	2,939 (2,382, 3,422)	<0.001	Treatment	299	0.286
		Control	2,719 (2,208, 3,063)	2,530 (2,134, 2,871)	<0.001	Time	588	0.037
		P (I vs. C)	0.579	0.001		Treatment × time	−367	0.039
	Relative vital capacity (ml/kg)	Intervention	44.16 (36.40, 52.48)	48.27 (42.31, 59.06)	<0.001	Treatment	8.21	0.111
		Control	46.22 (39.05, 52.58)	41.20 (35.27, 50.81)	<0.001	Time	13.6	0.009
		P (I vs. C)	0.823	0.823		Treatment × time	−8.33	0.011

### 3.3 Causal analysis

**Figure 2:**
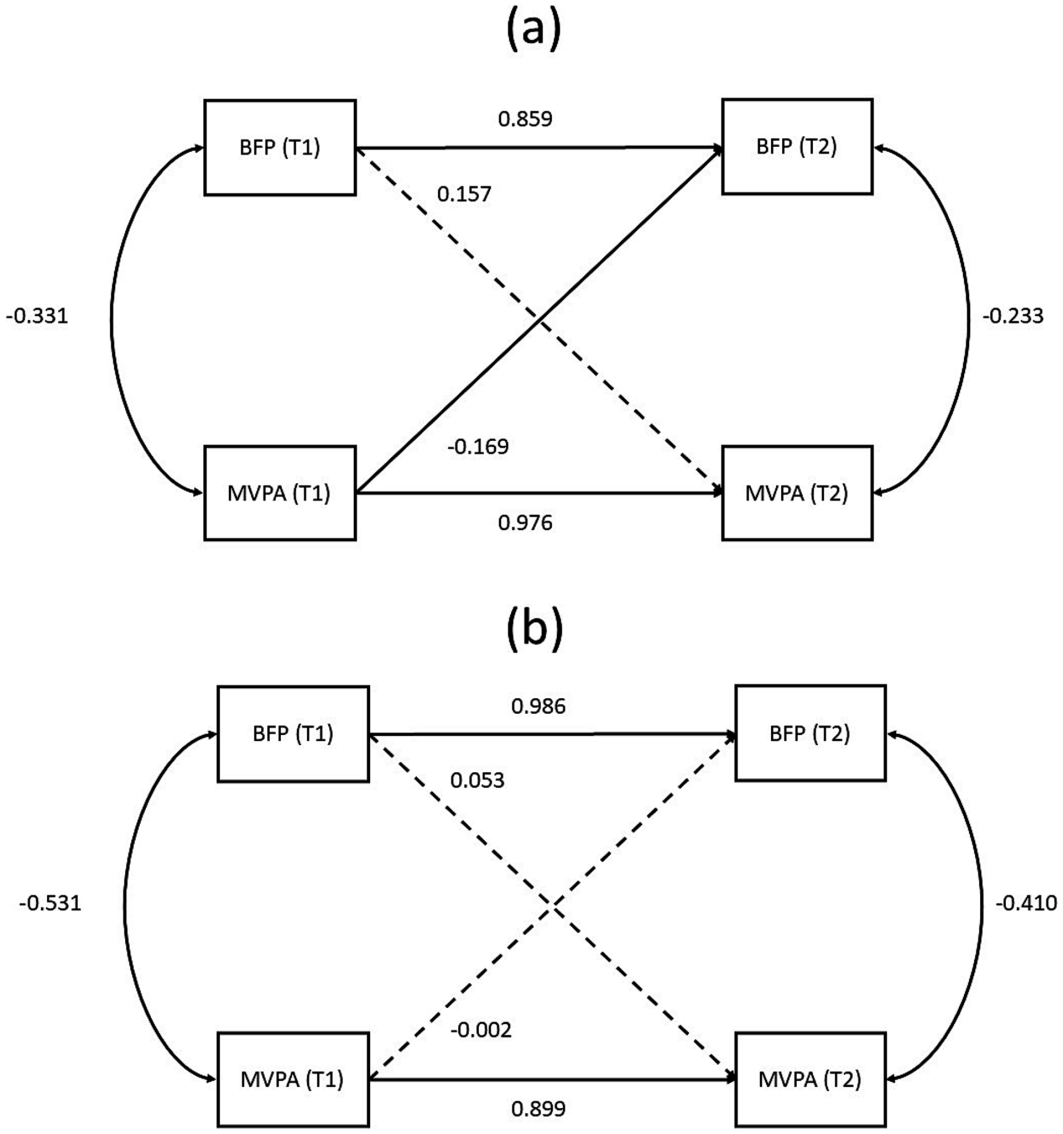
Cross-lagged panel model. Path diagrams illustrating the cross-lagged relationships between body fat percentage (BFP) and moderate-to-vigorous physical activity (MVPA) at baseline (T1) and after 12 weeks (T2) in **(a)** the intervention group and **(b)** the control group. Standardized path coefficients are shown. Solid lines represent significant paths (*p* < 0.05), indicating a predictive relationship, while dashed lines represent non-significant paths. The curved lines represent within-time correlations between BFP and MVPA.

We used cross-lagged panel models to examine the temporal relationships among body fat percentage (BFP), exercise self-efficacy (ESE), moderate-to-vigorous physical activity (MVPA), and self-esteem (SE). Initial models that included all four variables or any combination of three failed to achieve adequate model fit. Therefore, pairwise cross-lagged analyses were conducted. Only the model assessing the relationship between MVPA and BFP demonstrated acceptable fit in both the intervention and control groups (see Figure 2 for path diagrams; detailed fit indices are provided in Supplementary Table S2). In the intervention group (Figure 2a), both body fat percentage (BFP) and moderate-to-vigorous physical activity (MVPA) showed strong temporal stability over 12 weeks (BFP: *β* = 0.859; MVPA: *β* = 0.976). Importantly, there was a significant negative cross-lagged effect from baseline MVPA to follow-up BFP (*β* = −0.169, *p* < 0.05), indicating that higher MVPA at baseline predicted a greater reduction in BFP after the intervention. The reverse path from BFP to MVPA was not significant. Correlations between BFP and MVPA within each time point were negative but weakened slightly over time. In contrast, the control group (Figure 2b) demonstrated temporal stability for both measures, but no significant cross-lagged relationships emerged. Thus, only in the intervention group did higher baseline MVPA significantly predict improved body composition after 12 weeks.

## 4 Discussion

This study examined the SFR-OO. It focused on body composition, psychological factors, physical fitness, and exercise volume. The study explored the complex relationships between these factors. The intervention effectively reduced BFP, improved ESE, SE, and enhanced physical fitness, including knee push-ups, standing long jump, 4x10m shuttle run, and supine trunk lift time. However, no causal relationship was found between SE or ESE and physical activity, while physical activity significantly predicted BFP.

Results showed significant reductions in BFP in the intervention group, contrasting with an increase in the control group. After the intervention, the intervention group’s body fat was notably lower than the control group confirming the effectiveness of the intervention in managing body fat, consistent with prior studies (28). Edward P. Weiss et al. noted that weight loss through calorie restriction also reduces FFM (29). Exercise likely mitigated this loss in our study’s control group. While gender differences were not a focus, other studies suggest women may retain muscle better during calorie restriction (30).

The 12-week SFR-OO significantly enhanced SE and ESE, with greater improvements in the intervention group. This rise in SE aligns with findings on the positive impact of physical activity (28, 31), supporting the idea that successful exercise and weight loss boost SE (28, 32).

The SFR-OO significantly enhanced physical fitness in the intervention group, including knee push-ups, standing long jump, 4x10m shuttle run, and supine trunk lift time, with greater improvements than the control group. Our findings are consistent with previous research reporting the benefits of both aerobic and resistance training (33). However, there were no significant improvements in agility, coordination, or core strength. This likely reflects the program’s limited emphasis on these areas. Sports like basketball and tennis can improve agility (34). Future interventions should incorporate specific training to improve balance, reaction speed, and core strength (35). Research also suggests that motor coordination relates to physical activity, influenced by body fat (36). To more effectively enhance these aspects of physical fitness, future intervention programs should incorporate specialized training modules such as agility ladder drills, quick-foot exercises, and dynamic balance training, as well as core strength components including suspension training, plank exercises, and Pilates routines (37, 38). Therefore, this study highlights the need for more targeted and systematic training strategies aimed at improving agility, coordination, and core strength in adolescent health interventions, in order to achieve more comprehensive and effective outcomes. The study found no significant causal link between ESE and SE. These factors might not influence each other directly over a short period. Longer studies are needed to understand these dynamics (39). Also, no direct causal impact from ESE on MVPA was observed. MVPA is influenced by various factors beyond self-efficacy, such as environment and available time (40). Only in the intervention group did MVPA predict lower body fat. This aligns with other cross-sectional studies (41, 42). However, diet, genetics, age, and gender also affect body fat (43). Merely increasing MVPA might not explain all changes in body fat. The absence of significant causal relationships between psychological variables and behavioral or physiological outcomes may be attributed to several factors. First, changes in psychological factors often require a longer period to accumulate before resulting in observable behavioral modifications and physiological improvements (34, 44). Second, psychological and behavioral changes in adolescents are substantially moderated or constrained by family environment, peer influence, and the broader social-ecological context, which can attenuate the directly observable impact of psychological factors on behavioral and physiological outcomes (45). Future research should adopt longer follow-up periods and incorporate systematic assessment of social environment, family background, and peer relationships, in order to more accurately elucidate the complex interactions between psychological well-being, behavior, and physiological changes.

The initial cross-lagged models incorporating three to four variables (SE, ESE, MVPA, and BFP) did not achieve satisfactory model fit or explanatory power. Several plausible reasons could contribute to these inadequate model performances. First, the complexity and multifactorial nature of adolescent obesity likely involve numerous mediating or moderating factors not included in the present analysis—such as diet quality, peer influence, parental behaviors, school environment, and socioeconomic status—which can significantly impact psychological factors, physical activity, and body fat reduction simultaneously. Although this study controlled general dietary intake, nuanced dietary factors or individual dietary compliance variation may have impacted the outcomes. Additionally, psychological factors such as SE and ESE can be inherently dynamic and influenced by outside contextual determinants that are difficult to control in shorter-term interventions.

Although this study conducted innovative and empirically meaningful explorations in the areas of school-family-research integrated interventions and dynamic analyses of behavior and body fat, several major limitations should be acknowledged:

Intervention Duration and Psychological Effect Lag: The intervention and follow-up period in this study was 12 weeks, which allowed for preliminary observation of changes in MVPA and BFP. However, such a short duration may be insufficient to capture the medium- and long-term impact of psychological factors such as self-esteem and exercise self-efficacy on behavioral and physiological outcomes. As a result, the psychological effects pathway could not be fully elucidated. Future research should consider extending the intervention period and adding follow-up assessments to better evaluate the longitudinal effects of psychological, behavioral, and physiological interactions.

Sample Size and Multivariable Modeling Limitations: Although the sample size (*N* = 98) was appropriate for an initial intervention study, the statistical power is limited for modeling complex multivariable interactions and dynamic path relationships among variables such as SE, ESE, MVPA, and BFP. In particular, the cross-lagged models with multiple variables showed poor fit, making it difficult to robustly identify subtle but true causal pathways. This limitation also precluded robust sex-stratified or multi-center analyses, underscoring the need for larger samples and multicenter designs in future research.

Dietary Intervention and Control of Confounders: Dietary intervention in this study mainly relied on parental self-report, which is highly subjective and prone to recall bias and social desirability effects, thereby reducing the objectivity of dietary assessments. In addition, systematic assessment and control of confounding variables such as dietary compliance, family environment, and socioeconomic status were not sufficiently conducted. This may have affected the validity of some observed intervention effects and causal inferences.

Study Type and Generalizability: Overall, this study is best characterized as a pilot/exploratory randomized controlled trial (RCT). While it provides valuable insight into intervention effects and mechanistic pathways for subsequent research with larger samples, longer durations, and multi-center designs, the generalizability and causal extrapolation of the findings require further validation and expansion through large-scale, objective, and multi-dimensional research initiatives.

## 5 Conclusion

The 12-week SFR-OO successfully improved adolescents’ body composition, physical fitness, and psychology. The intervention positively influenced psychological measures, though its predictive power on physical outcomes remained limited. However, SE and ESE did not significantly predict MVPA or BFP. MVPA modestly predicted reduced body fat only within the intervention group, suggesting a context-dependent effect.

## Data Availability

The raw data supporting the conclusions of this article will be made available by the authors, without undue reservation.
