# Supplementary material for: Causal insights into the school-family-research integrated health promotion program for overweight and obesity: the independent role of moderate-to-vigorous physical activity in body fat reduction, undermined by psychological factors
Source: Front Nutr. 2025 Jul 29;12:1577319. doi: 10.3389/fnut.2025.1577319 (PMC12339317; doi:10.3389/fnut.2025.1577319)
Supplement: Supplementary file 1 [file Table_1.docx]

**Intervention Exercise Details**

1. **Core Flexors: Crunch**

**Band-assisted Crunch (Regression):** The resistance band is anchored above the head (e.g., to a fixed overhead point), with both hands gripping the band or the band looped across the shoulders. Lying supine with knees bent, the band provides upward traction during trunk flexion, reducing the activation threshold for the rectus abdominis. The lower back remains in contact with the floor, and the motion is controlled to prevent neck strain or hip flexor compensation.

**Standard Crunch (Standard):** The participant lies supine with knees flexed and feet flat on the floor, hands either alongside the head or crossed on the chest. Using abdominal contraction, the scapulae are lifted off the floor; the effort is focused on the rectus abdominis. Movement amplitude is moderate, a brief isometric hold may be performed at the top, and the trunk is lowered in a controlled manner.

**Band-resisted Crunch (Progression):** The resistance band is anchored to a low point behind the participant (e.g., the ground just behind the upper back), with both hands holding the band at the front of the shoulders (or the band crossed in front of the shoulders). When lifting the upper back, the participant must overcome the additional rear-directed tension created by the band, thus increasing core muscle activation. The lumbar spine remains in contact with the floor throughout, with a 1–2 second pause at the top of the movement. Movement should be free from compensatory strategies such as swinging or arm assistance.

1. **Upper Extremity Compound Push: Push-up**

**Inclined Push-up (Regression):** Hands are placed wider than shoulder-width on an elevated surface (bench, platform, or step). With the body in a straight line, the elbows are flexed to lower the chest toward the support, followed by extension to return. The inclined angle reduces the proportion of body weight borne by the arms and chest, facilitating skill acquisition.

**Kneeling Push-up (Regression):** From a quadruped position, the knees remain on the ground and the feet are lifted. The hands are placed shoulder-width apart. The participant performs a push-up with reduced resistance, lowering the chest until the elbows reach approximately 90°, then extending to return while maintaining spinal alignment and core engagement.

**Standard Push-up (Standard):** The participant assumes a prone plank position supported by hands and toes, hands positioned under the shoulders. With core and lower limb muscles contracted for stability, the body is lowered until the chest is just above the floor and the elbows approach a 90° angle. Arms then extend to bring the body back to the starting position. The movement must be performed with a neutral spine and without scapular winging.

**Band-resisted Push-up (Progression):** A resistance band loops across the upper back, with its ends fixed under the palms. The participant performs a standard push-up while overcoming additional upward resistance from the band, especially in the concentric phase. Proper control and spinal alignment must be sustained throughout.

1. **Lower Extremity Flexors: Nordic Hamstring Curl**

**Band-assisted Nordic Hamstring Curl (Regression):** The participant kneels on a padded surface with ankles securely fixed (e.g., under a bar), and a resistance band anchored above and looped over the torso or shoulders. The torso and thighs are kept rigid as the body slowly leans forward from the knees. The band provides assistance (reducing effective body weight load) during the descent. Hands may assist to safely return to the starting position.

**Standard Nordic Hamstring Curl (Standard/Progression):** The protocol is the same without a resistance band. The participant controls the forward eccentric movement as far as possible, ideally through the full available eccentric phase before using hands to control descent if necessary. The pelvis and trunk remain in neutral alignment during the entire range of motion.

1. **Core Extensors: Supine Back Extension**

**Standard Supine Back Extension (Standard):** The participant lies prone on a mat, with hands positioned at the sides of the head or extended along the body. By contracting the lumbar extensors and gluteal muscles, the upper trunk is elevated off the mat as far as flexibility and strength permit, followed by a slow and controlled return to the start. The cervical spine stays in line with the thoracic spine to avoid hyperextension.

**Band-resisted Back Extension (Progression):** A resistance band is anchored to the ground or a fixed low point. The band is looped across the upper thorax/back, so that trunk extension occurs against the additional downward tension of the band, requiring increased muscle activation. Ensure the lumbar region remains neutral and that the motion is slow and controlled at all times.

1. **Upper Extremity Compound Pull: Pull-up**

**Band-assisted Low Bar Pull-up (Regression):** Performed on a bar lower than the participant’s standing reach, with a resistance band looped over the bar and around the feet. The band’s upward tension assists the participant during elbow flexion and shoulder adduction, reducing the intensity required from the upper limb and back muscles. The core is braced, and momentum is avoided.

**Low Bar Pull-up (Regression/Intermediate):** With a low bar, the participant’s feet can lightly contact the ground, offloading a fraction of body weight. The participant pulls the chin over the bar while maintaining controlled technique, then lowers with control.

**Band-assisted Standard Pull-up (Intermediate):** On a full-height pull-up bar, a resistance band is looped over the bar, with the feet or knees in contact with the band. The band provides substantial assistance, enabling proper pull-up technique for those not yet able to perform the exercise unaided.

**Standard Pull-up (Progression):** The participant performs a dead hang grip on the bar, engaging the scapular stabilizers first, then actively flexing the elbows and adducting the shoulders to pull the chin above the bar. The descent (eccentric phase) is performed slowly and with control, without excessive swinging.

1. **Upper Extremity Extensors: Band-resisted Shoulder Press**

**Band-resisted Shoulder Press (Standard/Progression):**The resistance band is anchored under the participant’s feet, and both hands hold the band at shoulder height with palms facing forward. The participant presses upward until elbows approach full extension overhead (without full elbow lockout), then lowers back to the shoulders in a controlled fashion. Core activation is essential to prevent lumbar hyperextension throughout the motion.

1. **Lower Body Functional Movement: 50-meter Sprint Intervals**

**50-meter Sprint Intervals:**The participant performs maximal effort sprints over a 50-meter distance, followed by recovery (walking or slow jogging) back to the start. Multiple repetitions are performed per session as prescribed. Technical emphasis is placed on explosive initiation, coordinated arm swing, and efficient stride mechanics. After each sprint, adequate active/complete recovery enables quality speed efforts and minimizes injury risk.

1. **Lower Extremity Extensors: Squat**

**Band-assisted Squat (Regression):** One end of the resistance band is anchored above (e.g., doorframe or high horizontal bar), with both hands gripping the band. Standing with feet shoulder-width apart, the participant squats down while the band provides upward assistance throughout the descending and ascending phases, thereby reducing the load borne by the lower limbs. The posture should be maintained with a neutral spine, knees aligned with the toes, and core engaged to prevent lumbar flexion or extension.

**Standard Squat (Standard):** Performed without external assistance, with feet shoulder-width apart and toes slightly externally rotated. The movement consists of simultaneous hip and knee flexion, lowering the thighs to parallel or just below parallel to the ground, while maintaining a straight back and neutral pelvis. The participant then extends the hips and knees to return to standing. Weight should remain distributed across the whole foot; control and tempo should be prioritized.

**Unilateral/Band-resisted Single-leg Squat (Progression):** Performed as a pistol squat or single-leg squat, either unaided or with an added resistance band anchored under the standing foot or at the side for increased intensity. While standing on one leg with the opposite leg extended forward or backward, the participant squats to the lowest controllable point, focusing on stability and alignment. The movement is initiated and controlled by the hip extensors, gluteals, and quadriceps; core stabilization is paramount throughout the movement to prevent pelvic tilt or trunk rotation.

**Methods for Modified Physical Fitness Test Protocols**

**Knee Push-ups**

The knee push-up is a youth-adapted modification of the Eurofit protocol that emphasizes upper body and core muscular endurance. Before starting, the participant places hands on the floor slightly wider than shoulder-width, with both knees and hands supporting their weight while the feet are lifted off the ground. Throughout the assessment, the body should form a straight line from the head through the hips to the knees, with the core engaged to avoid sagging or elevating the hips. During each repetition, the elbows are flexed to lower the chest as close as possible to the floor—without touching—then fully extended to return to the starting position. The maximum number of correct repetitions is recorded within one minute or until voluntary exhaustion. Improper repetitions (e.g., insufficient depth, loss of alignment, incomplete arm extension) are excluded from the final count. If the participant fails to reach the standard for two consecutive repetitions, the test may be terminated early. The examiner monitors form closely and the test must be stopped immediately if the participant feels pain or discomfort.

**Supine Trunk Lift Time**

The supine trunk lift time adapts the Eurofit protocol to assess trunk extensor endurance rather than flexibility or static strength. The participant lies supine on a mat, with arms crossed on the chest or behind the head and legs either bent or straight according to comfort. At the examiner’s signal, the participant contracts the back extensor muscles, slowly raising the upper back and scapulae off the mat to their maximum attainable height, and then maintains this position. Timing starts once the peak raised position is achieved and the posture is stabilized. The participant must hold this position as long as possible, maintaining a neutral head and spine alignment and avoiding compensatory movements. Timing ends if the upper back or scapulae touch the mat, the raised posture is lost, compensatory movements are observed, or the subject voluntarily lowers the trunk. The total holding time (in seconds) is recorded as the result. The examiner supervises throughout to ensure technical correctness and to prevent injury, stopping the test immediately if the participant experiences any back pain or discomfort.
