# Supplementary material for: Causal insights into the school-family-research integrated health promotion program for overweight and obesity: the independent role of moderate-to-vigorous physical activity in body fat reduction, undermined by psychological factors
Source: Front Nutr. 2025 Jul 29;12:1577319. doi: 10.3389/fnut.2025.1577319 (PMC12339317; doi:10.3389/fnut.2025.1577319)
Supplement: Supplementary file 2 [file Table_2.docx]

| **Group** | **Model** | **p-value** | **χ²/df** | **GFI** | **RMSEA** | **RMR** | **CFI** | **NFI** | **NNFI** |
| --- | --- | --- | --- | --- | --- | --- | --- | --- | --- |
| Intervention | MVPA ↔ BFP | 0.260 | 2.930 | 0.980 | 0.058 | 0.013 | 0.999 | 0.997 | 0.999 |
| Control | MVPA ↔ BFP | 0.370 | 0.805 | 0.997 | 0.010 | 0.043 | 1.000 | 0.997 | 0.996 |

**Table S2. Goodness-of-Fit Indices for Cross-lagged Panel Models of MVPA and BFP**

Abbreviations: GFI, goodness-of-fit index; RMSEA, root mean square error of approximation; RMR, root mean square residual; CFI, comparative fit index; NFI, normed fit index; NNFI, non-normed fit index.
